# Diagnostic Dilemma: Ocular Features and Treatment Outcome of a Case of Coats-Like Retinitis Pigmentosa

**DOI:** 10.7759/cureus.71695

**Published:** 2024-10-17

**Authors:** Suyi Siow, Nurul Ashikin Abdullah

**Affiliations:** 1 Ophthalmology, Hospital Kuala Lumpur, Kuala Lumpur, MYS

**Keywords:** anti-vegf treatment, coats' disease, cystoid macular edema, intermediate uveitis, retinitis pigmentosa (rp)

## Abstract

Coats-like retinitis pigmentosa is a rare disease demonstrating both features of Coats disease and retinitis pigmentosa. We are reporting a case of a 15-year-old female with no known medical illness who presented with a one-year history of nyctalopia and bilateral painless blurred vision. Fundus examination revealed bilateral optic disc swelling, telangiectasia, and bony spicules. Optical coherence tomography and fundus fluorescein angiography showed cystoid macular edema (CMO).

The diagnosis of Coats-like retinitis pigmentosa with CMO was made. She received laser indirect ophthalmoscope photocoagulation and intravitreal anti-vascular endothelial growth factor therapy, but her vision remained refractory. Her diagnosis was subsequently revised to bilateral intermediate uveitis causing CMO and her vision improved to periocular steroid injections. This case emphasizes how critical it is to identify symptoms and diagnose the illness as soon as possible because treating related complications on time can save a patient's sight and provide long-term benefits.

## Introduction

Retinitis pigmentosa (RP) is a group of inherited retinal disorders that is characterized by progressive degeneration of photoreceptors (mainly rods) and retinal pigment epithelium (RPE). It can be inherited in an autosomal recessive, autosomal dominant, or X-linked manner. Coat’s disease is an idiopathic retinal vasculopathy, distinguished by telangiectatic vessels, aneurysm, and intraretinal and subretinal lipid exudation. Zamorani first reported a single patient demonstrating features of both RP and Coat’s disease in 1956 [1]. There were then subsequent case reports and case series describing patients with both diseases hence the term “Coats-like RP” was proposed. The prevalence of Coats-like RP approximates 5% of all RP patients [1-3]. We present a case of Coats-like RP whereby the patient demonstrates both clinical features of RP and exudative retinopathy with cystoid macula edema (CMO) that remains refractory to multiple intravitreal anti-vascular endothelial growth factor therapy (anti-VEGF) injections.

## Case presentation

**Figure 1:**
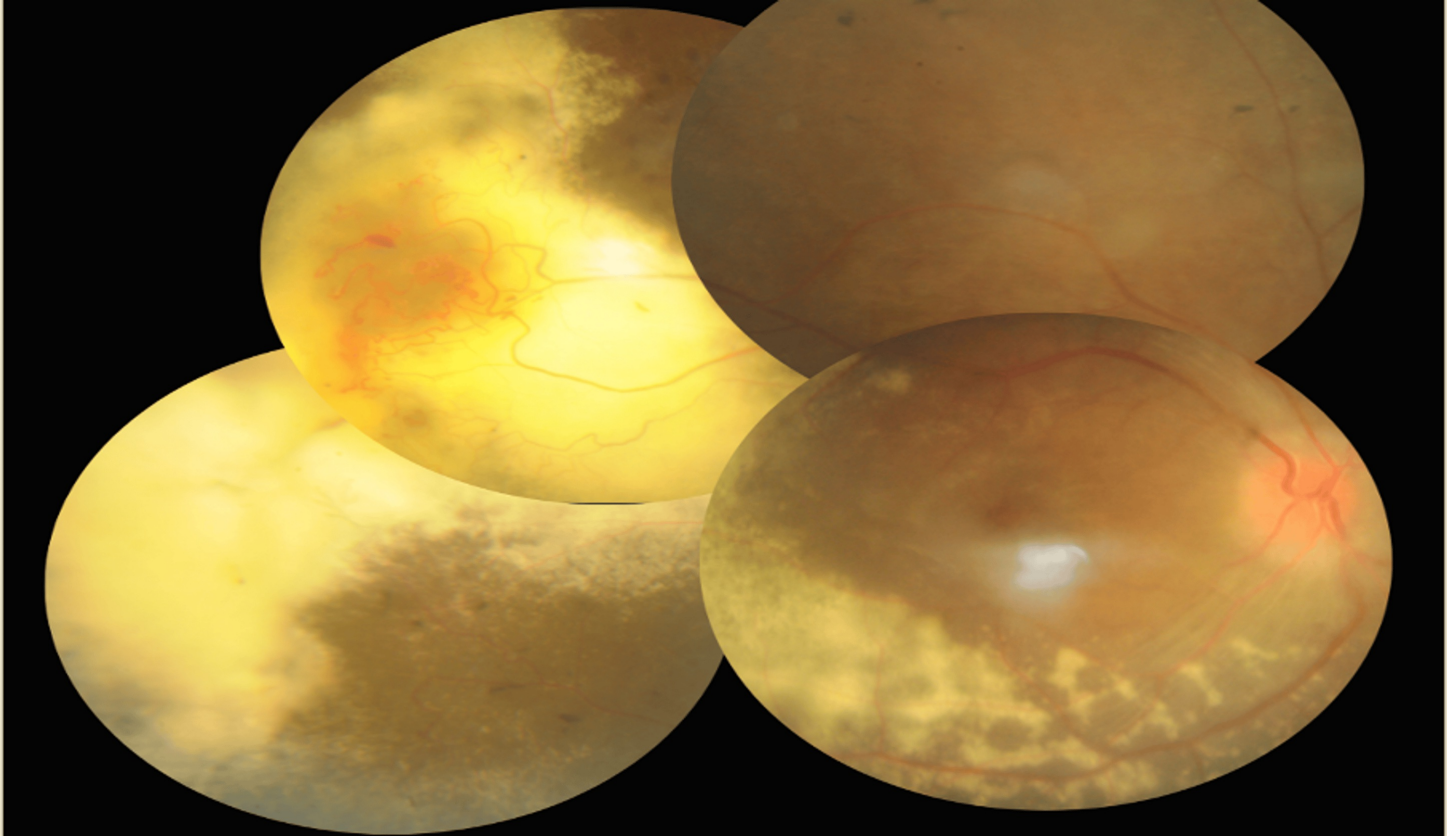
Right eye fundus

**Figure 2:**
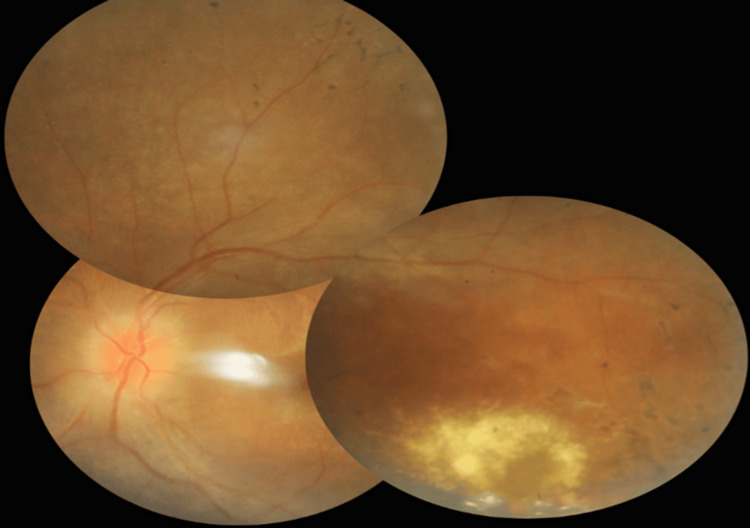
Left eye fundus

A 15-year-old girl with no known medical illness presented to the ophthalmology department with a one-week history of painless bilateral blurred vision associated with right eye central scotoma. This episode was preceded by a one-year history of nyctalopia whereby she had difficulty seeing objects in dim light and at nighttime. Otherwise, she has no history of eye redness, trauma, floaters, or photopsia. She has no family history of retinal disease. On examination, her best-corrected distance visual acuities were 6/36 respectively. Near vision was N10 over the right eye and N12 over the left eye. The right fundus (Figure 1) demonstrated a swollen and hyperemic optic disc and extensive hard exudates with telangiectatic vessels temporally. The left fundus (Figure 2) also showed a swollen optic disc, hard exudates inferotemporal, and hyperpigmented bony spicules at the peripheral retina.

**Figure 3:**
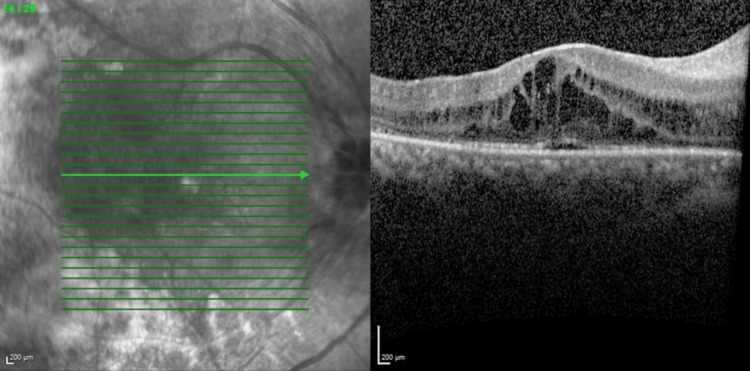
Optical coherence tomography macula showing right eye cystoid macula edema with photoreceptor disruption parafoveally (central retinal thickness =570µm)

**Figure 4:**
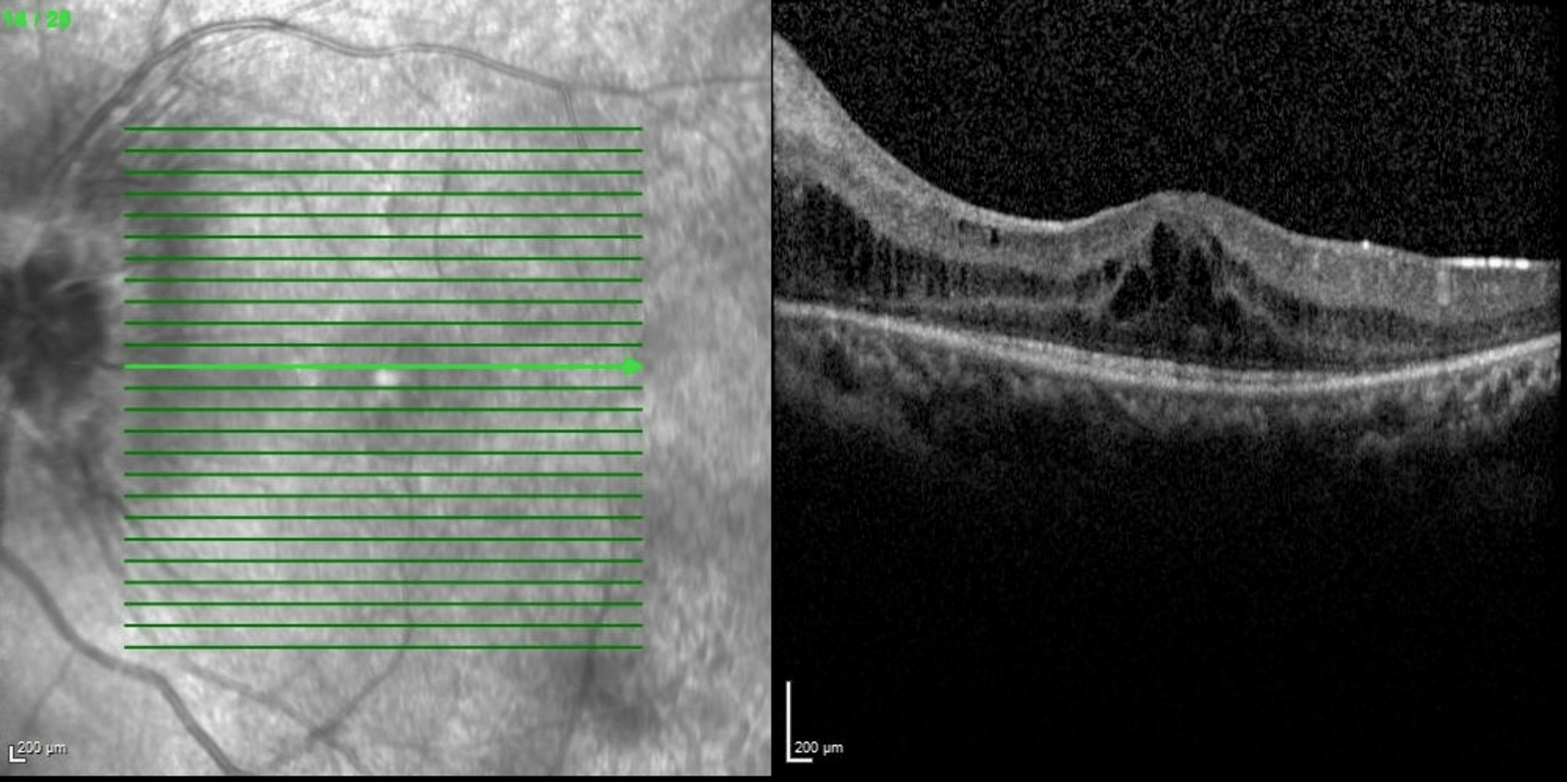
Optical coherence tomography macula showing left eye cystoid macula edema with photoreceptor disruption parafoveally (central retinal thickness=556µm)

Optical coherence tomography (OCT) macula (Figures 3, 4) showed bilateral CMO with photoreceptor disruption parafoveally and epiretinal membrane. Fundus fluorescein angiography (FFA) showed hot disc, telangiectatic vessels with an area of capillary nonperfusion peripherally, and angiographic CMO over bilateral eyes and generalized leakages over the left eye. Electroretinogram (ERG) revealed a reduction in a and b waves with poorer scotopic response compared to photopic response.

A diagnosis of Coat’s like RP with CMO was made. In view of her young age, the patient received laser indirect ophthalmoscope photocoagulation and anti-VEGF under general anesthesia for the treatment of telangiectatic lesions. She was initially started on systemic carbonic anhydrase inhibitor to treat CMO, however, was then switched to intravitreal anti-VEGF injection in view of no improvement.

**Figure 5:**
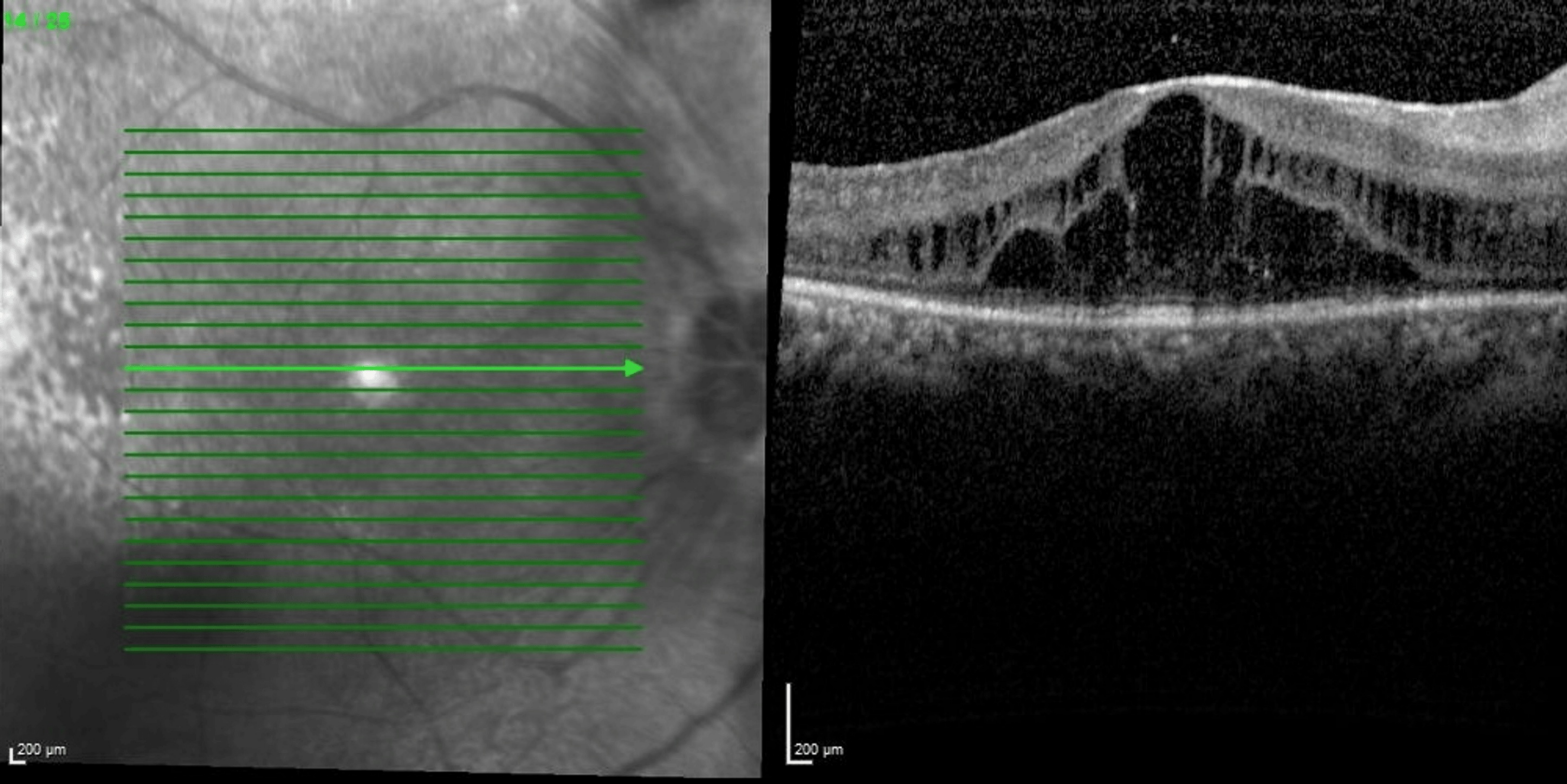
Optical coherence tomography of right eye macula after three doses of intravitreal ranibizumab (central retinal thickness=758µm)

**Figure 6:**
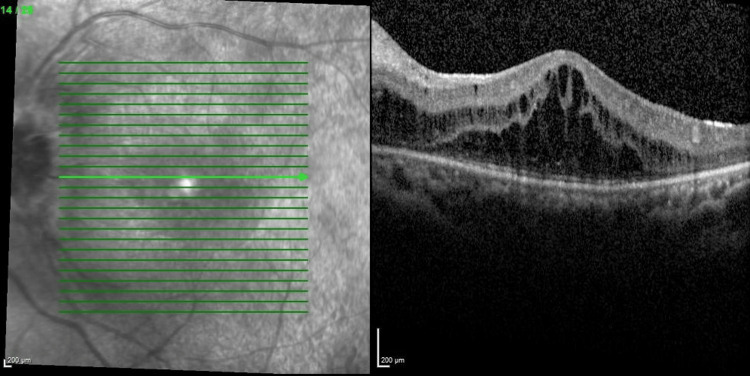
Optical coherence tomography of left eye macula showing refractory CMO after three doses of intravitreal ranibizumab (central retinal thickness=627µm)

**Figure 7:**
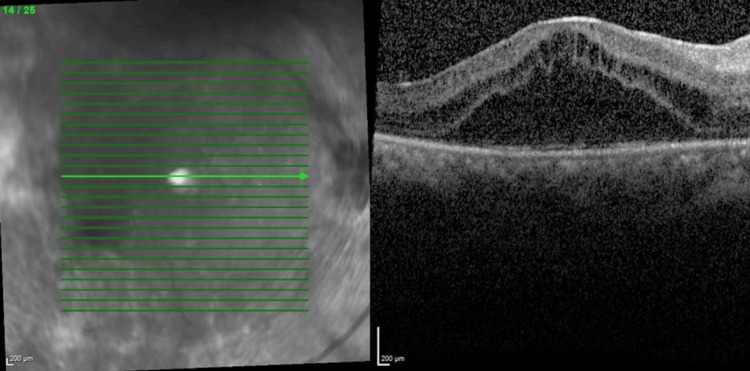
Optical coherence tomography of right eye macula after four doses of intravitreal aflibercept (central retinal thickness=558µm)

**Figure 8:**
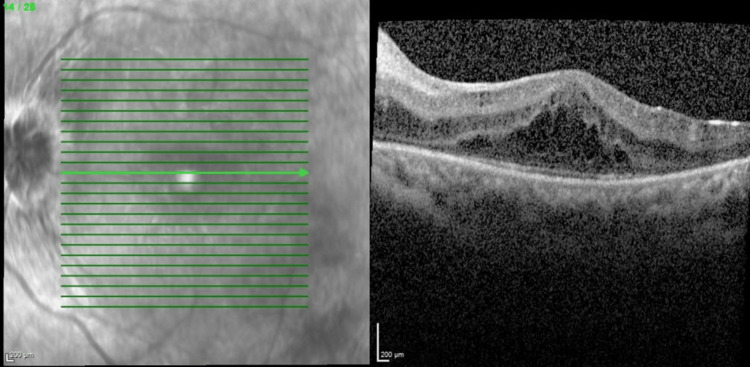
Optical coherence tomography of left eye macula after four doses of intravitreal aflibercept (central retinal thickness=473µm)

Her progress throughout multiple intravitreal anti-VEGF injections is shown in the figures below. The decision to reinject relies on the patient's visual acuity and central retinal thickness (CRT) on the OCT macula. Her bilateral vision was 6/36 at the initial presentation. She was commenced with bilateral intravitreal ranibizumab injection at six to eight weeks intervals. Despite three doses of intravitreal ranibizumab, her vision and CMO remained refractory as shown in Figures 5, 6. The decision was then made to switch to intravitreal aflibercept injection, and it was similarly given at six to eight weeks intervals. There was a slight improvement in her vision, but the CRT remained static even after four injections as shown in Figures 7, 8.

**Figure 9:**
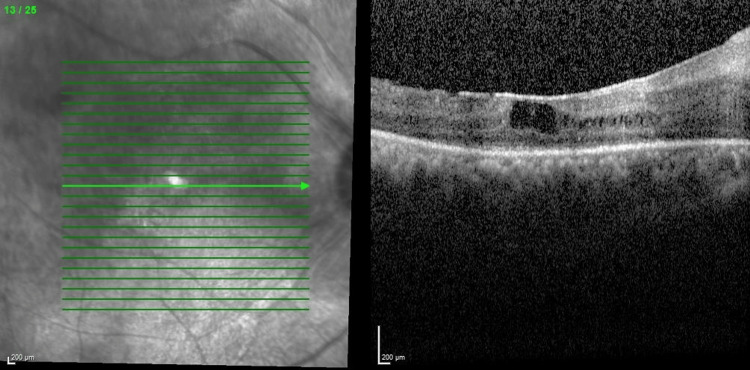
Optical coherence tomography of right eye macula after subtenon triamcinolone showing reduction in central retinal thickness (central retinal thickness=311µm)

**Figure 10:**
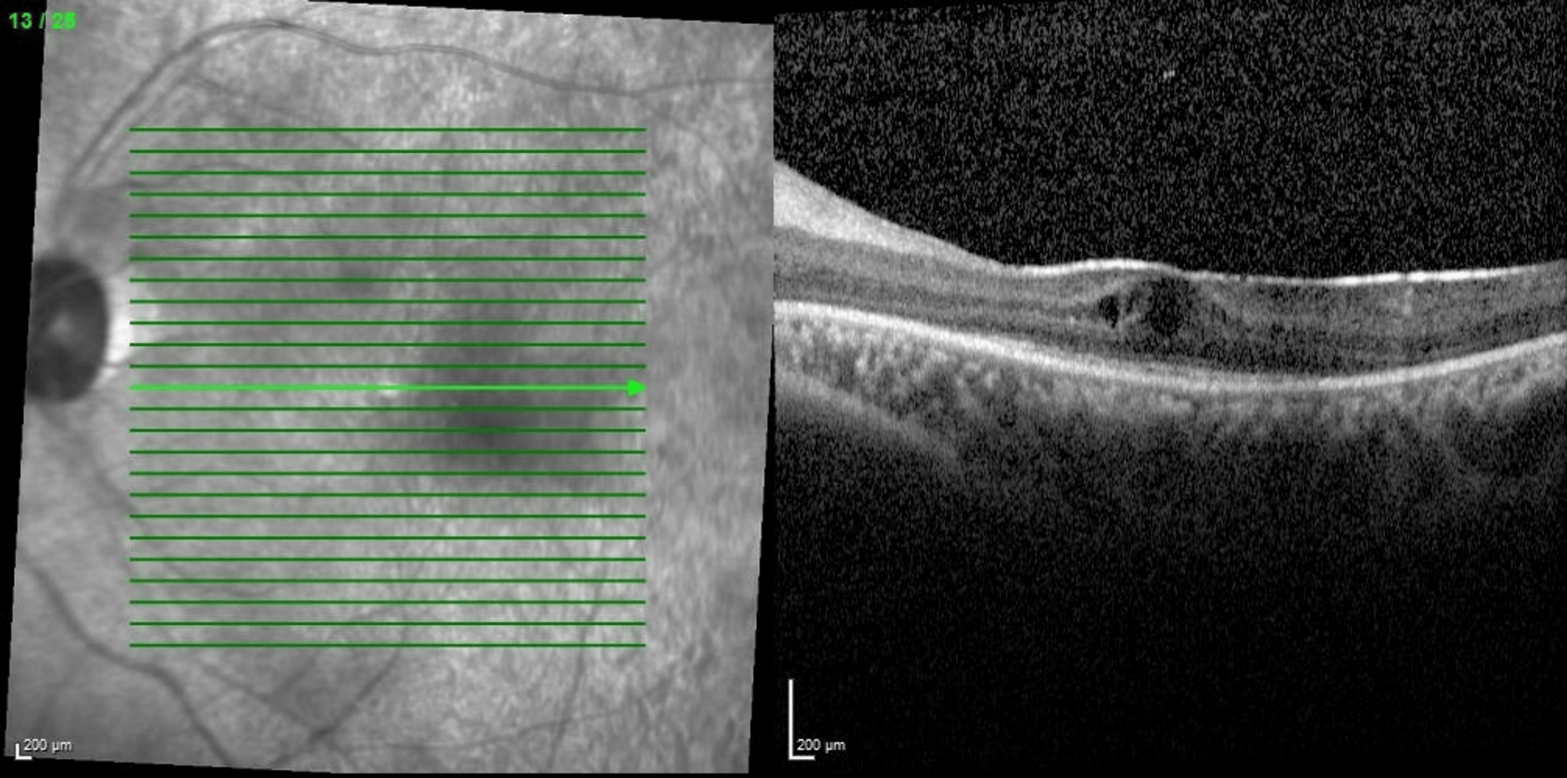
Optical coherence tomography of left eye macula after subtenon triamcinolone showing reduction in central retinal thickness (central retinal thickness=292µm)

The other possibility of persistent CMO was considered and eventually attributed to a revised diagnosis of bilateral intermediate uveitis (IV) as there was evidence of anterior vitreous cells and vitritis during the subsequent follow-up. A uveitic workup performed ruled out infective causes. She was then started with an immunosuppressive dose of systemic steroid with concurrent immunomodulator and bilateral superotemporal subtenon triamcinolone injection was done in view of the persistent CMO. Finally, she responded well with periocular steroid injection, tapering dose of systemic steroid and immunomodulator and her distance vision improved to 6/12 bilaterally with a reduction of central retinal thickness as evident in Figures 9, 10. Near vision also improved to N6 in both eyes.

## Discussion

RP is the most common inherited retinal dystrophy and has an estimated prevalence of one in 4,000 worldwide. Patients often develop nyctalopia before losing peripheral field vision and eventually progress to legal blindness by middle age. The disease predominantly affects the rod photoreceptors before impacting the cones in later stages [2,4].

Coats disease is classically a unilateral disease, manifesting in more male than female patients in late childhood or adolescence. It is rarely associated with RP, classically presenting in young males unilaterally, and characterized by a triad of telangiectatic vessels, dilated aneurysms, and lipid exudation [1]. Compared to isolated Coats disease, retrospective studies have shown that Coats-like RP has no predilection for males but demonstrated equal female and male incidence. The timing of onset ranges from childhood to late adulthood with a mean age of onset in the fourth decade. It is commonly bilateral in presentation. Lesions are typically located at the inferior and temporal retina as evident in our patient. In a recent multicenter study in which 67 Coats-like RP patients were included, it was reported that a combination of lipid depositions, telangiectatic vessels, and retinal detachment was the commonest presentation. Exudative RD was found in 63% of patients in this study [5]. On the other hand, the incidences of CMO have been reported to be widely variable in different studies, occurring in 26%-50% of eyes. The cause of CME is presumed to be secondary to the disruption of blood blood-retinal barrier and reduction of the RPE pumping mechanism [6].

Management of Coats-like RP includes observation, laser treatment, anti-VEGF, cryotherapy, pars plana vitrectomy, and scleral buckle. With respect to managing CMO, laser photocoagulation, systemic or topical acetazolamide, anti-VEGF, and steroid injection have all demonstrated subtle to substantial reduction in central retinal thickness. We have likewise started the patient on oral acetazolamide and intravitreal anti-VEGF therapy. It is postulated that intravitreal anti-VEGF works by negating the effect of VEGF in mediating inflammation and vascular endothelial cell permeability in the subretinal layer.

In this case nonetheless, despite multiple anti-VEGF sessions, the patient’s vision remains static. She was subsequently found to develop anterior vitreous cells in both eyes whereby a diagnosis of bilateral IU was made. We believe that this is the first reported case of bilateral IU in a case of coats-like RP. Even to date, the literature has very limited case reports of intermediate uveitis in RP. Badawi reported a case IU in a young RP patient associated with a novel homozygous splice site mutation in PRPF8. The study demonstrated that a short course of topical steroids can be an effective treatment in which there was the resolution of IU [7]. As the patient was refractory to multiple anti-VEGF posing a therapeutic challenge, sub-tenon triamcinolone (STTA) was subsequently given to both eyes to which she responded extremely well. There was a near-total resolution of intermediate uveitis and CMO.

## Conclusions

We are highlighting this case as a differential diagnosis to RP as this can be sight-changing with earlier diagnosis and intervention of its treatable complications. Further studies with larger patient populations are required to explore the genetics and long-term visual prognosis of this variant of RP. Also, the revision on the cause of CMO should be considered in cases of refractory to multiple intravitreal anti-VEGF injections as shown in this case.
